# Improving the prediction of disease-related variants using protein three-dimensional structure

**DOI:** 10.1186/1471-2105-12-S4-S3

**Published:** 2011-07-05

**Authors:** Emidio Capriotti, Russ B Altman

**Affiliations:** 1 Department of Bioengineering, Stanford University, Stanford CA, USA; 2 Department Genetics, Stanford University, Stanford CA, USA; 3 Department of Mathematics and Computer Science, University of Balearic Islands, Palma de Mallorca, Spain

## Abstract

**Background:**

Single Nucleotide Polymorphisms (SNPs) are an important source of human genome variability. Non-synonymous SNPs occurring in coding regions result in single amino acid polymorphisms (SAPs) that may affect protein function and lead to pathology. Several methods attempt to estimate the impact of SAPs using different sources of information. Although sequence-based predictors have shown good performance, the quality of these predictions can be further improved by introducing new features derived from three-dimensional protein structures.

**Results:**

In this paper, we present a structure-based machine learning approach for predicting disease-related SAPs. We have trained a Support Vector Machine (SVM) on a set of 3,342 disease-related mutations and 1,644 neutral polymorphisms from 784 protein chains. We use SVM input features derived from the protein’s sequence, structure, and function. After dataset balancing, the structure-based method (SVM-3D) reaches an overall accuracy of 85%, a correlation coefficient of 0.70, and an area under the receiving operating characteristic curve (AUC) of 0.92. When compared with a similar sequence-based predictor, SVM-3D results in an increase of the overall accuracy and AUC by 3%, and correlation coefficient by 0.06. The robustness of this improvement has been tested on different datasets and in all the cases SVM-3D performs better than previously developed methods even when compared with PolyPhen2, which explicitly considers in input protein structure information.

**Conclusion:**

This work demonstrates that structural information can increase the accuracy of disease-related SAPs identification. Our results also quantify the magnitude of improvement on a large dataset. This improvement is in agreement with previously observed results, where structure information enhanced the prediction of protein stability changes upon mutation. Although the structural information contained in the Protein Data Bank is limiting the application and the performance of our structure-based method, we expect that SVM-3D will result in higher accuracy when more structural date become available.

## Background

In recent years, though the cost of genomic experiments has decreased rapidly, the interpretation of their results is still an open problem. The complete sequencing of the human genome in 2003 [1] led to the identification of millions of Single Nucleotide Polymorphisms (SNPs) by the HapMap Consortium project [2] and the Human Variation project [3]. This created a significant need for bioinformatics tools to analyze the large amount of data to detect functional SNPs and describe their molecular effects. Currently the number of validated SNPs in the dbSNP database is greater than 20 million [4]. In general, mutations occurring in coding regions may have a greater impact on the gene’s functionality than those occurring in non-coding regions [5]. Only a small fraction of SNPs (~60,000) corresponds to the subset of annotated missense coding SNPs [6]. For this subset of Single Amino acid Polymorphisms (SAPs), curators of the Swiss Institute of Bioinformatics classify them into disease-related SAPs and neutral SAPs, according to the corpus of peer-reviewed literature. In the last few years, several methods have been developed to predict the impact of a given SAP [7-26]. These algorithms are able to predict the effect of the mutation on protein stability [13,14,19,23-26], protein functional activity [9,21], and insurgence of human pathologies [7,8,10-12,15-18,20-22]. The majority of methods rely on information derived from protein sequence [7,11,12,17], while others use protein structure data [8,15,20,22,27-29] and knowledge-based information [10,16,18]. In particular, SIFT [30] and PolyPhen2 [18] rely on different representations of evolutionary information. SIFT scores the normalized probabilities for all possible substitutions at a mutated site using a multiple sequence alignment of homologous proteins. PolyPhen2 predicts the impact of variants by calculating a Position Specific Independent Counts (PSIC) matrix from a multiple sequence alignment. Protein family specific HMM models have also been implemented in PANTHER [7] to detect deleterious mutations. Machine learning-based approaches such as PhD-SNP [12] and SNAP [9] have shown better results with respect to traditional approaches. Recently described methods rely on knowledge-based information to reach overall accuracy greater than 80%. For instance, SNPs&GO [10] includes a new log-odd score calculated from GO terms, and MutPred [16] uses different machine learning approaches to evaluate the probabilities of gain or loss of predicted structural and functional properties. The structural context of the mutations has been studied to determine the mechanism of action of each mutation at the protein level [8]. In addition, protein three-dimensional structural features have been used to improve the prediction of the impact of SAPs on protein function [21] and human health [22,31]. Although the predictive power of protein structural information has been established, a quantitative comparison between structure-based and sequence-based methods is still needed. In this paper, we focus our attention on the prediction of disease-related SAPs using a novel machine learning-based method that takes as input, protein sequence, protein structure, and protein function information (SVM-3D). For the fist time, we predict deleterious single point mutations considering in a unique framework protein structure information, used for the prediction of stability changes in I-Mutant [13,23], and protein sequence, evolutionary and functional information, used in the recently developed SNPs&GO algorithm [10]. To quantify the improvement of the performance resulting from the use of protein structure information, we compared the accuracy of SVM-3D against a similar sequence and function-based method (SVM-SEQ) [10], SIFT [30] and PolyPhen2 [18]. In particular, the comparison with PolyPhen2 [18] is more appropriate because it considers in input structural features such as secondary structures, solvent accessibility and normalized B factor of the mutated residue. The results show that protein three-dimensional structure information increases the accuracy in detection of deleterious SAPs and can provide insight about the disease mechanism.

## Results

### Performance of the method

**Figure 1:**
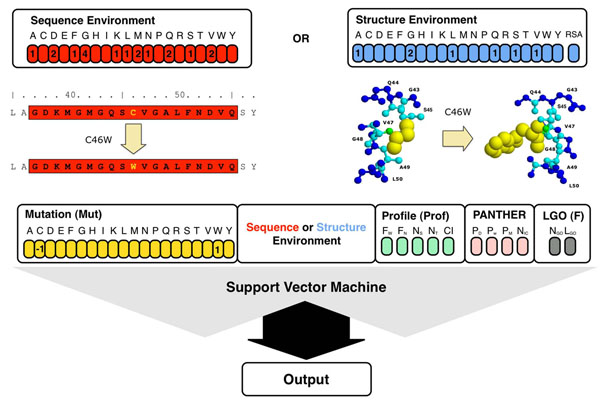
Flow chart of our SVM-based methods. The structure-based method (SVM-3D) takes in input mutation (yellow) structure environment (in blue), sequence profile (green), PANTHER output (pink) and function (gray) information. In the sequence-based method (SVM-SEQ) the 21 elements vector encoding for the structural environment is replaced by the 20 elements vector encoding for the sequence environment. The structure environment is the residue composition in a 6 Å radius shell around the C-a of the mutated residue. The sequence environment is the amino acid composition window of 19 residues centred on the mutated residue.

**Figure 2:**
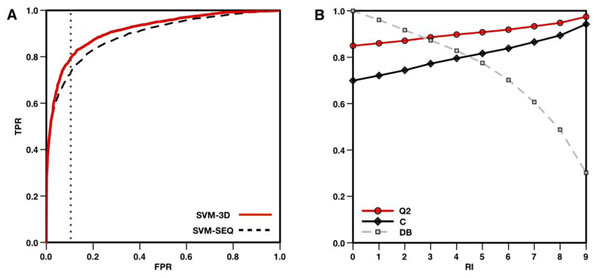
Performance of the structural-based method. In panel (A), ROC curves of the sequence (SVM-SEQ) and structure-based methods (SVM-3D). The plot shows the improvement of 3% in AUC and 7% in TPR when sequence and structure base methods are compared. In panel B, accuracy and correlation coefficient of SVM-3D as function of the Reliability Index (RI). If predictions with RI>5 are selected the SVM-3D method results in 91% overall accuracy 0.82 correlation coefficient over 78% of the dataset. Accuracy measures (Q2, C, TPR and FPR) are defined in Methods section. DB is the fraction of the whole dataset of mutations.

In the last decades, machine learning approaches have been successfully used to address several biological problems and develop new prediction tools. We modified a previously developed predictor [10] by introducing three-dimensional structure information. In particular, we used new features to describe the structural environment of the mutation, examining the protein elements within a radius of 6 Å around the C-α atom (see Figure 1). To quantify the improvement in accuracy resulting from the use of 3D structure information, we compared the performance of our structure-based method (SVM-3D) with a sequence-based one (SVM-SEQ). In Table 1 are reported different accuracy measurements for both predictors tested on B3D dataset (see Methods). The structure-based method results in 3% improvement in overall accuracy and 0.06 higher correlation. Comparing the ROC curves (Figure 2 A), SVM-3D gives 0.03 better Area Under the Curve (AUC) with respect to SVM-SEQ. If 10% of wrong predictions are accepted, SVM-3D has 7% more true positives. The output returned by the SVM has been used to calculate the Reliability Index (RI) in order to filter predictions. If predictions with RI>5 are selected, the SVM-3D method achieves 91% overall accuracy and 0.82 correlation coefficient on 78% of the whole dataset (see Figure 1 B). Analyzing the predictions of SVM-SEQ and SVM-3D, we found that the outputs agree in the 91% of the cases. On this subset, the overall accuracy is 87% and the correlation coefficient of the method is 0.74. For the remaining 9% of the predictions, SVM-SEQ results in very poor overall accuracy and correlation, 34% and -0.32, respectively. SVM-3D performs slightly better than random, giving 66% overall accuracy and 0.32 correlation coefficient (see Table 2).

**Table 1:** Performances of the sequence (SVM-SEQ) and structure (SVM-3D) based methods.

	Q2	P[D]	S[D]	P[N]	S[D]	C	AUC
SVM-SEQ	0.82	0.81	0.83	0.82	0.81	0.64	0.89
SVM-3D	0.85	0.84	0.87	0.86	0.83	0.70	0.92

The accuracy measures are defined in Methods section. D and N are disease-related and neutral variants respectively. Methods are tested on the B3D dataset.

**Table 2:** Performances on agree and not agree subset of predictions

	Q2	P[D]	S[D]	P[N]	S[D]	C	AUC	PM
SEQ∩3D	0.87	0.85	0.89	0.89	0.84	0.74	0.93	91
SEQ-3D	0.66	0.70	0.70	0.62	0.62	0.32	0.71	9
3D-SEQ	0.34	0.38	0.30	0.30	0.38	-0.32	0.35	9

SEQ∩3D indicates the subset of agree predictions between SVM-3D and SVM-SEQ. SEQ-3D and 3D-SEQ are respectively the predictions of SVM-SEQ and SVM-3D on the not agree prediction subset. The accuracy measures are defined in Methods section. PM is the fraction of the dataset. D and N are disease-related and neutral variants respectively.

### Comparison with other methods

The accuracy of our SVM-based methods has been compared with SIFT and PolyPhen2. To score the performance of the methods on a set composed with highly reliable neutral polymorphism, we calculated their accuracy on N3D dataset. The results in Table 3 show that SVM-3D has 2% higher accuracy and 3% higher correlation coefficient with respect to the PolyPhen2 and SVM-SEQ. In addition SVM-3D and SIFT result in 79% sensitivity in the prediction of neutral polymorphism that is 3% higher than the same value reached by SVM-SEQ and PolyPhen2. Although the level of improvement is slightly lower with respect to previously reported data, we should note that it has been obtained only on ~12% of the whole A3D dataset.

**Table 3:** Comparison with other methods on the N3D dataset.

	Q2	P[D]	S[D]	P[N]	S[D]	C	AUC	PM
SIFT	0.77	0.77	0.74	0.76	0.79	0.53	0.83	96
PolyPhen2	0.80	0.78	0.83	0.82	0.76	0.60	0.86	99
SVM-SEQ	0.80	0.78	0.84	0.82	0.76	0.59	0.86	100
SVM-3D	0.82	0.80	0.85	0.84	0.79	0.63	0.89	100

The accuracy measures are defined in Methods section. D and N are disease-related and neutral variants respectively. PM is the predicted fraction of the dataset. Methods are tested on N3D dataset.

To evaluate the minimum level of improvement resulting from the use of protein structure information, we performed a more stringent test using a set of mutations (KIN) occurring in proteins annotated with the Gene Ontology term “Kinase activity” (GO:0016301). Our SVM-based methods have been trained on a dataset (noKIN) without any protein with “Kinase Activity” and without any significant sequence similarity to proteins in KIN dataset. To keep the number of disease-related and neutral polymorphism balanced, in the training step the neutral variants have been doubled considering their reverse mutations. We have compared the performances of our sequence (SVM-SEQ) and structure-based (SVM-3D) methods against SIFT and PolyPhen2. The scores obtained on KIN dataset confirm that SVM-3D results in 3% higher accuracy and more than 3% correlation with respect to SIFT and PolyPhen2 (see Table 4). These values represent a significant lower bound level of improvement (probability c^2^ ≤ 0.01) since in the noKIN training set there is not any protein with significant sequence similarity (e-value >10^-3^) to proteins in KIN dataset. In addition, all functional information associated to GO:0016301 and its sub annotations are not considered for the calculation of the LGO score in the KIN dataset. In this test, performed on a lower number of mutations, although the improvement of the performances between SVM-SEQ and SVM-3D is not significant (probability c^2^ = 0.25), SVM-3D is still resulting in 2% higher accuracy and 0.03 higher correlation coefficient with respect to SVM-SEQ.

**Table 4:** Comparison with other methods on the KIN dataset.

	Q2	P[D]	S[D]	P[N]	S[D]	C	AUC	PM
SIFT	0.80	0.90	0.84	0.58	0.69	0.50	0.81	99
PolyPhen2	0.80	0.88	0.86	0.60	0.63	0.48	0.81	98
SVM-SEQ	0.81	0.87	0.88	0.63	0.62	0.50	0.82	100
SVM-3D	0.83	0.87	0.91	0.69	0.59	0.53	0.83	100

The accuracy measures are defined in Methods section. D and N are disease-related and neutral variants respectively. PM is the predicted fraction of the dataset. Methods are tested on KIN dataset.

### Structure environment analysis

**Figure 3:**
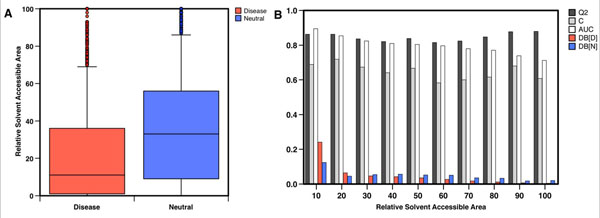
Analysis of the protein three-dimensional structure environment. In panel (A) the distribution of the relative solvent accessible area (RSA) for disease-related and neutral variants. The significant difference of their distributions makes the RSA a good feature to discriminate between disease-related and neutral variants. In panel (B) we report the accuracy of SVM-3D predictions as a function of the RSA. The plot shows that the accuracy of SVM-3D is lower in exposed regions with respect to buried ones. Accuracy measures (Q2, C and AUC) are defined in Methods section. DB is the fraction of the whole dataset for disease-related (D) and neutral (N) mutations.

Protein three-dimensional structural information is an important feature for predicting the effects of SAPs. Analysis of the protein structure provides information about the environment of the mutation. In fact, the effect of a mutation depends critically upon the location of the residue, specifically if it is buried in the hydrophobic core or exposed on the surface of the protein. In Figure 3 panel A, we plot the distributions of the relative solvent accessible area (RSA) for disease-related and neutral variants. The two distributions have mean RSA values of 20.6 and 35.7 for disease-related and neutral variants, respectively (see Figure 3 panel A). They are significantly different and the Kolmogorov-Smirnov test results in a p-value of 2.8·10^-71^. We calculated the overall accuracy and correlation coefficient of our method dividing the dataset in 10 bins according to RSA value of the mutated residue. The SVM-3D method shows better performance in the prediction of buried (RSA<20) and highly exposed (RSA>80) mutated residues (see Figure 3 panel B).

### Scoring the residue interactions

**Figure 4:**
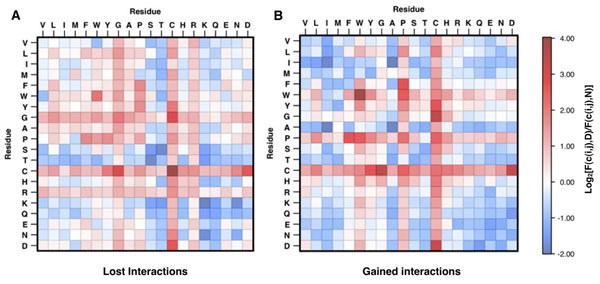
Log-odd score for lost residues interactions (A) and for gained interactions (B). The red and blue zones correspond to damaging and neutral interactions respectively. The mutations resulting in the lost of a Cys-Cys and the gain of Trp-Trp interactions are mainly associated to insurgence of disease.

Protein structure gives insight to the interactions between residues far in primary sequence but close in 3D space. We defined two types of interactions: the “lost” interactions are those missing as a direct result of the mutation event and the “gained” interactions are those expected to be formed by the new (mutant) residue. We compared the frequency of lost and gained interactions in the context of disease or neutral mutations. In Figure 4 panels A and B, we show the log-odd scores for lost and gained interactions, respectively. According to these results, the most deleterious lost contacts are between cysteines (Cys-Cys) and the most damaging gained interactions are between tryptophans (Trp-Trp). A missing Cys-Cys interaction can lead to the loss of a disulphide bond that strongly contribute to the protein’s stability by modulating the hydrophobicity of both native and denatured states [32]. The mutation of a residue to a tryptophan when close to other aromatic residues can stabilize the structure but may increase the protein aggregation rate [33].

**Figure 5:**
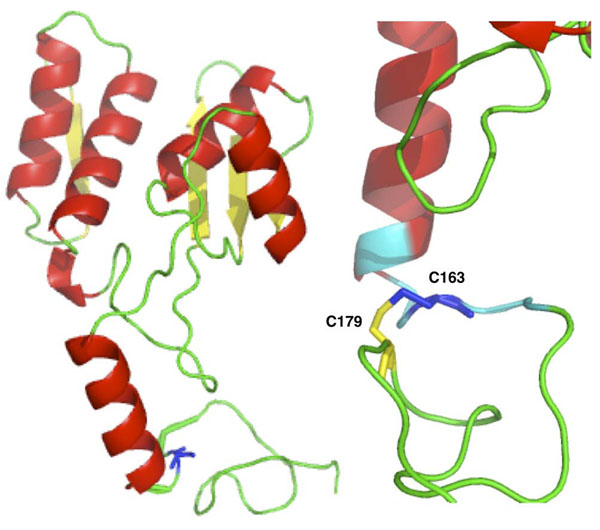
Structure of the Glycosylasparaginase (PDB code 1APY chain A) and details of the region around Cys163 (in blue). Residues in cyan and Cys179 (yellow) are below 6 Å. Residues 163 and 179 are numbered respectively 140 and 159 in the PDB file.

**Figure 6:**
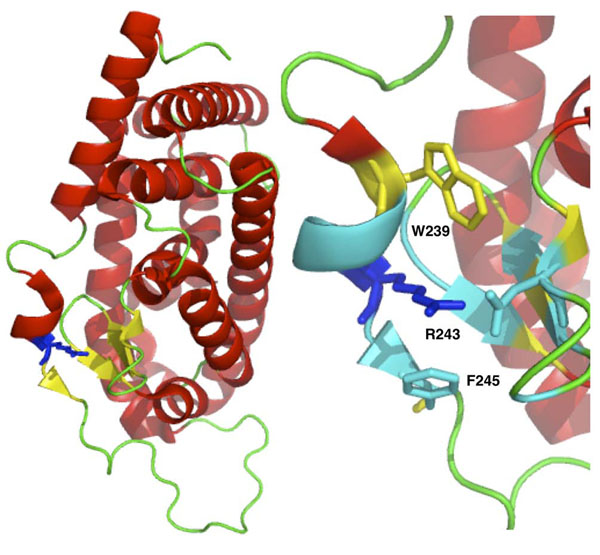
Structure of the Thyroid hormone receptor (PDB code 1NAX chain A) and details of the region around the Arg243 (blue). Residues in cyan and Trp239 (yellow) are below 6 Å.

We discuss two examples where sequence-based method wrongly classifies two disease-related variants while structure-based algorithm is able to predict them correctly. An example of lost Cys-Cys interaction is the mutation of Cys163 in Glycosylasparaginase (Swiss-Prot:ASPG_HUMAN). This mutation is responsible for the insurgence of Aspartylglucosaminuria (MIM:208400). Visual inspection of the protein structure (Figure 5) shows that mutation of Cys163 to Serine results in the loss of the disulfide bridge between Cys163 and Cys179 (respectively Cys140 and Cys156 in the PDB structure 1APY chain A). An interesting example of a possibly damaging gained interaction can be observed in the Thyroid hormone receptor (Swiss-Prot:THB_HUMAN), where mutation of Arg243 to tryptophan is cause of Thyroid hormone resistance (MIM:188570,274300). Analyzing the protein structure (1NAX chain A), we expect that the new tryptophan will be in proximity to another tryptophan in position 239 and a phenylalanine in position 245. Thus, this mutation could result in stereo-chemical problems in the pocket around the position 243 (see Figure 6). In addition after mutation, the 3 aromatic residues (Trp239, Trp243, Phe245) in the exposed region could increase aggregation rate of the protein. The case of Cys163Ser variant in Glycosylasparaginase is a good example where structure-based method SVM-3D results in correct prediction because it able to capture the disulfide bond between Cys163 and Cys179 that is not described by the local sequence environment represented in SVM-SEQ.

## Discussion

The results of this work show that protein structure information increases the accuracy of the prediction of deleterious mutations. The increments of 3% in overall accuracy and AUC, and 6% in correlation coefficient, with respect to the sequence-based method, are comparable with the improvement of the performance obtained using protein function information [10]. Although this gain is significant (probability c^2^ = 4·10^-7^), it is not as high as expected. In the next future, higher number of mutations from proteins with known structure will increase the performances of structure-based methods with respect to sequence-based ones. The Protein Data Bank (PDB) only contains a subset of structures describing mutant proteins and the quaternary interactions. Due to these limitations, our method only takes in to account structural information about the wild-type protein and intra-chain tertiary interactions. This model is a good approximation to describe the structural environment of the buried residues but it is not appropriate for exposed residues. This limitation justifies the lower level of accuracy observed for exposed residues (see Figure 3B). In particular, for mutated residues with more than 40% exposed surface the correlation coefficient of the predictions is lower than the mean correlation coefficient resulting from the sequence-based predictor. The limitations of our algorithm to describe the structural changes and the environment of exposed residues make the gain of 3% in accuracy and AUC a lower bound estimation of the improvement. Similar level of improvement has been obtained in the stringent test of KIN dataset where, without “Kinese Activity” functional information and homolog proteins in the training set, our structure-based method resulted in ~3% higher accuracy and AUC with respect to SIFT and PolyPhen2. These results are particularly encouraging because the SVM-3D method reaches higher level of accuracy with respect to PolyPhen2, which includes protein structure information in the input features. According to these observations, further improvements of our structure-based method will require the knowledge of the three-dimensional structure of the mutated proteins and the protein-protein interactions.

## Conclusion

We developed a new machine learning approach that integrates protein structure information to predict the effects of SAPs. To quantify the increase in accuracy achieved by protein structure information, we compared our method to a previously developed sequence-based predictor. Using a balanced set of 6,630 mutations, the structure-based method results in about 3% higher accuracy and AUC and 0.06 higher correlation with respect to the sequence-based approach. In addition, our SVM-3D approach results in 3% better accuracy and AUC with respect to SIFT and PolyPhen2. Although the increase in performance is not extremely high, the introduction of structure information provides insight about disease mechanism. The prediction improvement is also in agreement with previous results, where structure information enhanced the prediction of protein stability change upon amino acid mutation [13].

## Methods

### Datasets

The performance of machine learning methods strongly depends on the training set. Thus, the selection of a representative and unbiased set of SAPs is an important issue in the development of predictive algorithms. A previous analysis of different SAPs databases has shown annotated variants from the Swiss-Var database to be the best [34]. According to this observation, we selected our set of SAPs from Swiss-Var release 57.9 (Oct 2009) and then mapped the variants to protein structures from the Protein Data Bank (PDB) [35]. We used a precompiled list of correspondences between Swiss-Prot and PDB codes available at the ExPASY web site. Using this mapping, we aligned each pair of sequences using the BLAST algorithm [36] and then filtered out alignments with: i) gaps, ii) sequence identity lower than 100%, and iii) shorter than 40 residues. The remaining alignments were used to calculate the correspondence between the Swiss-Prot and PDB residue numerations. In the case where a mutation mapped to more than one protein structure, the structure with best resolution was used. After this filtering procedure, we obtained a set of 4,986 mutations from 784 protein chains (A3D). Specifically, this corresponds to 3,342 disease-related SAPs and 1,644 neutral polymorphisms. To keep the dataset balanced, we doubled the number of neutral variants by considering their reverse mutation as neutral. The final dataset (B3D) was therefore composed by 6,630 mutations, about equally distributed between disease-related and neutral SAPs. The performance of our algorithm has been evaluated considering a well characterized subset of neutral polymorphism mapped on dbSNP database and with minor allele frequency higher than 0 in the three main populations (CEU, YRI and HCB/JPT). This subset consists of 311 neutral mutations annotated as with higher reliability. To perform this second test, we build the N3D dataset that is composed by previously selected 311 neutral polymorphisms and the same number of randomly selected deleterious mutations from A3D dataset.

To estimate the lower level of improvement in the prediction performance, resulting from using structural information, we performed another test selecting the subset of mutations occurring in proteins annotated with the “Kinase activity” Gene Ontology term (GO:0016301). This dataset (KIN) is composed by 492 mutations in 75 protein chains 369 of which are annotated as disease-related and the remaining 123 as neutral polymorphisms. The performances of the method are evaluated training our machine learning approach on the remaining set of mutations corresponding to proteins not annotated with GO:0016301 term. To make the test more stringent, we also removed from the training set those mutations occurring in proteins which have one BLAST hit to KIN proteins with e-value lower than 10^-3^. After this procedure, the final training set of non-kinase mutations (noKIN) is composed by 4,379 mutations from 671 protein chains of which 2,919 disease-related and 1,460 neutral polymorphisms. The composition of the datasets used in this work is summarized in Table 5 and all data are available in the Additional file 1.

**Table 5:** Composition of the datasets

	Total	Disease	Neutral	PDB Chains
A3D	4,986	3,342	1,644	784
B3D	6,630	3,342	3,288	784
N3D	622	311	311	328
KIN	492	369	123	75
noKIN	4,379	2,919	1,460	671

### Implemented SVM-based predictors

The addressed task is to predict whether a given single amino acid polymorphism is neutral or disease-related. The task is defined as a binary classification problem for the protein undergoing mutation. The Support Vector Machine (SVM) input features for the structure-based predictor include the amino acid mutation, the mutation’s structural environment, the sequence-profile derived features, and the functional-based log-odds score calculated from the GO classification terms (see Figure 1). The final input vector consisted of 52 elements:

• 20 components encoding for the mutations (Mut)

• 21 features representing local protein structure (Structure Environment)

• 5 features derived from sequence profile (Prof)

• 4 features from the output of PANTHER method (PANTHER)

• 2 elements encoding the number of GO terms associated to the protein and the GO log-odd score (LGO).

A similar sequence-based SVM predictor has been used to measure the increase in accuracy stemming from the use of protein three-dimensional structure information [10]. The structure-based SVM differs only in the 21 elements encoding for the local protein structure environment (Structure Environment). These replace the 20 elements encoding for the sequence environment used by the sequence-based SVM predictor (see Figure 1).

#### Encoding residue mutation

The input vector relative to mutation consists of 20 values corresponding to the 20 residue types. It explicitly defines the mutation by setting the element corresponding to the wild-type residue to -1 and the newly introduced residue to 1 (all the remaining elements are kept equal to 0).

#### Encoding mutation structure environment

The protein structural environment is encoded with a 21 elements vector. The first 20 features encode the count for each residue type proximal to the mutated residue. Proximal residues must have at least one heavy atom within a given distance of the C-α atom of the mutated residue. After an optimization procedure, a distance cutoff of 6 Å was selected. The 21^st^ element is the relative solvent accessible area (RSA) calculated using the DSSP program [37]**.**

#### Encoding mutation sequence environment

The 20 elements input values for the mutation sequence environment match the 20 residue types. They track the occurrence of each residue type in proximity in primary sequence to the mutated residue. Included positions are those found inside a window centered on the mutated residue and that symmetrically spans the sequence to the left (N-terminus) and to the right (C-terminus) with a total length of 19 [12].

#### Encoding sequence profile information

We derived for each mutation the sequence profile, comprising: the frequency of the wild-type (*F_W_*), the frequency of the mutated residue (*F_N_*), the number of totally (*N_T_*) and locally aligned sequences (*N_S_*), and a conservation index (*CI*) [38] for the position at hand. The conservation index is calculated as:

(1) *CI*(*i*)=*[Σ_a_*_=_*_1_^20^*(*f_a_*(*i*)*-f_a_*)*^2^]^1/2^*

where *f_a_*(*i*) is the relative frequency of residue *a* at mutated position *i* and *f_a_* is the overall frequency of the same residue in the alignment. The sequence profile is computed from the output of the BLAST program [36] run on the uniref90 database (Oct 2009) (E-value threshold=10^-9^, number of runs=1).

#### PANTHER features

The 4 elements vector from PANTHER [39] output is composed by the probability of deleterious mutation (*P_D_*), the frequencies of the wild-type (*P_W_*) and new (*P_N_*) residues in the PANTHER family alignment and the number of independent counts (*N_IC_*). In case that PANTHER does not return any output the *P_D_* is set to 0.5 and the remaining value have been set to 0.

#### Functional based score

The Gene Ontology log-odds score (LGO) provides information about the correlation among a given mutation type (disease-related and neutral) and the protein function. LGO score was previously introduced to annotate cancer or non-cancer gene sets [40]. Recently, this log-odd score has been extended and used to distinguish between disease-related and neutral genes [10]. For each GO term, the frequency of mutants in the disease-related subset was compared to that in the neural subset and the log-odds score was calculated. The annotation data are relative to the GO Database (version Mar 2010) and are retrieved at the web resource hosted at European Bionformatics Institute (EBI). To calculate the LGO, first we derived the GO terms from all three branches (molecular function, biological process and cellular components) for all our proteins in the dataset. For each annotated term the appropriate ontology tree has been used to retrieve all the parent terms with the GO-TermFinder tool (http://search.cpan.org/dist/GO-TermFinder/) [41]. Each GO term has been counted only once. The log-odds score associated to each protein is calculated as:

(2) *LGO*=*Σ_GO_ log_2_[f_GO_*(*D*)*/f_GO_*(*N*)*]*

where *f_GO_* is the frequency of occurrence of a given GO term for the disease-related (D) and neutral mutations (N) adding one pseudo-count to each class. To prevent overfitting, the LGO scores are evaluated considering f_GO_ values computed over the training sets without including in the GO term counts of the corresponding test set.

#### Support Vector Machine software

The LIBSVM package (http://www.csie.ntu.edu.tw/~cjlin/libsvm/) has been used for the SVM implementation [42]. The selected SVM kernel is a Radial Basis Function (RBF) kernel K(x_i_,x_j_)=exp(-γ||x_i_-x_j_||^2^) and γ and C parameters are optimized performing a grid like search. After input rescaling the values of the best parameters are C=8 and γ=0.03125

### Statistical indexes for accuracy measure

The performances of our methods are evaluated using a 20-fold cross-validation procedure on the whole SAPs dataset. The dataset has been divided keeping the ratio of the disease-related to the neutral polymorphism mutations similar to the original distribution of the whole set. To avoid the presence of homolog proteins in both training and testing sets, all the proteins in the datasets are clustered according to their sequence similarity with the *blastclust* program in the BLAST suite [36] by adopting the default value of length coverage equal to 0.9 and the percentage similarity threshold equal to 30%. We kept all the mutations belonging to a protein in the same training set to avoid overestimation of the performance. In this paper the efficiency of our predictors have been scored using the following statistical indexes.

The overall accuracy is:

(3) *Q2*=*P/T*

where *P* is the total number of correctly predicted class of mutations and *T* is the total number of mutations. The Matthew’s correlation coefficient *C* is defined as:

(4) *C*(*s*)=*[p*(*s*)*n*(*s*)*-u*(*s*)*o*(*s*)*] / W*

where *W* is the normalization factor:

(5) *W*=*[*(*p*(*s*)+*u*(*s*))(*p*(*s*)+*o*(*s*))(*n*(*s*)+*u*(*s*))(*n*(*s*)+*o*(*s*))*]^1/2^*

for each class *s* (D and N, stand for disease-related and neutral mutations respectively); *p*(*s*) and *n*(*s*) are the total number of correct predictions and correctly rejected assignments, respectively, and *u*(*s*) and *o*(*s*) are the numbers of false negative and false positive for the class *s*.

The coverage *S* (sensitivity) for each discriminated class *s* is evaluated as:

(6) *S*(*s*)=*p*(*s*)*/[p*(*s*)+*u*(*s*)*]*

where p(s) and u(s) are the same as in Equation 5.

The probability of correct predictions *P* (or positive predictive values) is computed as:

(7) *P*(*s*)=*p*(*s*) */ [p*(*s*) + *o*(*s*)*]*

where *p*(*s*) and *o*(*s*) are the same as in Equation 5 (ranging from 0 to 1).

For each prediction a reliability score (*RI*) is calculated as follows:

(8) *RI*=20*abs |O(*D*)*-0.5|*

where *O*(*D*) ranges from 0 to 1 and it is the probability associated to the class disease-related (D) returned when LIBSVM is run with the probability estimation option. Other standard scoring measures, such as the area under the ROC curve (AUC) and the true positive rate (TPR= Q(s)) at 10% of False Positive Rate (FPR= 1-P(s)) are also computed [43].

### Interaction score

The residues interactions are defined considering all the residues within a radius shell of 6 Å around the C-α of the mutated residue. According to this we calculate a log odd score dividing the frequency of lost interactions related to disease by the same type of interactions that have no pathological effect.

Although the mutations could be responsible for protein structural changes, as first approximation, we consider the position of the C-α of the new residue will not change significantly after the mutation. Hence, we consider gained interactions those between the mutant residue and the residues previously interacting with the wild-type. The score of the possible damaging effect of interactions is computed as follow

(9) *LC*=*log_2_[f*(*c*(*i*,*j*),*D*)*/f*(*c*(*i*,*j*),*N*)*]*

where *f_k_*(*c*(*i*,*j*),*D*) and f(c(i,j),N) are the frequencies of contacts between residues *i* and *j* respectively for disease-related (D) and neutral (N) variants. The *LC* score has been calculated both for lost and gained interactions and are available in the Additional file 2 and 3 respectively.

## Authors' contributions

EC carried out the computational analysis. EC and RBA conceived and designed the study as well as drafted the manuscript. All authors read and approved the final manuscript.

## Competing interests

The authors declare that they have no competing interests.

## Supplementary Material

Additional file 1 List of Single Amino Acid Polymorphisms in our datasets. Click here for file

Additional file 2 Log-odd scores for lost residues pair interactions. Click here for file

Additional file 3 Log-odd scores for gained residues pair interactions. Click here for file

## References

[B1] International Human Genome Sequencing Consortium Finishing the euchromatic sequence of the human genome Nature 2004 431 7011 931 945 10.1038/nature03001 15496913

[B2] HapMap Consortium A haplotype map of the human genome Nature 2005 437 7063 1299 1320 10.1038/nature04226 16255080 PMC1880871

[B3] Cotton RG Auerbach AD Axton M Barash CI Berkovic SF Brookes AJ Burn J Cutting G den Dunnen JT Flicek P GENETICS. The Human Variome Project Science 2008 322 5903 861 862 10.1126/science.1167363 18988827 PMC2810956

[B4] Sherry ST Ward MH Kholodov M Baker J Phan L Smigielski EM Sirotkin K dbSNP: the NCBI database of genetic variation Nucleic Acids Res 2001 29 1 308 311 10.1093/nar/29.1.308 11125122 PMC29783

[B5] Cargill M Altshuler D Ireland J Sklar P Ardlie K Patil N Shaw N Lane CR Lim EP Kalyanaraman N Characterization of single-nucleotide polymorphisms in coding regions of human genes Nat Genet 1999 22 3 231 238 10.1038/10290 10391209

[B6] Yip YL Famiglietti M Gos A Duek PD David FP Gateau A Bairoch A Annotating single amino acid polymorphisms in the UniProt/Swiss-Prot knowledgebase Hum Mutat 2008 29 3 361 366 10.1002/humu.20671 18175334

[B7] Thomas PD Kejariwal A Campbell MJ Mi H Diemer K Guo N Ladunga I Ulitsky-Lazareva B Muruganujan A Rabkin S PANTHER: a browsable database of gene products organized by biological function, using curated protein family and subfamily classification Nucleic Acids Res 2003 31 1 334 341 10.1093/nar/gkg115 12520017 PMC165562

[B8] Wang Z Moult J SNPs, protein structure, and disease Hum Mutat 2001 17 4 263 270 10.1002/humu.22 11295823

[B9] Bromberg Y Yachdav G Rost B SNAP predicts effect of mutations on protein function Bioinformatics 2008 24 20 2397 2398 10.1093/bioinformatics/btn435 18757876 PMC2562009

[B10] Calabrese R Capriotti E Fariselli P Martelli PL Casadio R Functional annotations improve the predictive score of human disease-related mutations in proteins Hum Mutat 2009 30 8 1237 1244 10.1002/humu.21047 19514061

[B11] Capriotti E Arbiza L Casadio R Dopazo J Dopazo H Marti-Renom MA Use of estimated evolutionary strength at the codon level improves the prediction of disease-related protein mutations in humans Hum Mutat 2008 29 1 198 204 10.1002/humu.20628 17935148

[B12] Capriotti E Calabrese R Casadio R Predicting the insurgence of human genetic diseases associated to single point protein mutations with support vector machines and evolutionary information Bioinformatics 2006 22 22 2729 2734 10.1093/bioinformatics/btl423 16895930

[B13] Capriotti E Fariselli P Casadio R I-Mutant2.0: predicting stability changes upon mutation from the protein sequence or structure Nucleic Acids Res 2005 33 Web Server issue W306 310 15980478 10.1093/nar/gki375 PMC1160136

[B14] Guerois R Nielsen JE Serrano L Predicting changes in the stability of proteins and protein complexes: a study of more than 1000 mutations J Mol Biol 2002 320 2 369 387 10.1016/S0022-2836(02)00442-4 12079393

[B15] Karchin R Diekhans M Kelly L Thomas DJ Pieper U Eswar N Haussler D Sali A LS-SNP: large-scale annotation of coding non-synonymous SNPs based on multiple information sources Bioinformatics 2005 21 12 2814 2820 10.1093/bioinformatics/bti442 15827081

[B16] Li B Krishnan VG Mort ME Xin F Kamati KK Cooper DN Mooney SD Radivojac P Automated inference of molecular mechanisms of disease from amino acid substitutions Bioinformatics 2009 25 21 2744 2750 10.1093/bioinformatics/btp528 19734154 PMC3140805

[B17] Ng PC Henikoff S Predicting deleterious amino acid substitutions Genome Res 2001 11 5 863 874 10.1101/gr.176601 11337480 PMC311071

[B18] Ramensky V Bork P Sunyaev S Human non-synonymous SNPs: server and survey Nucleic Acids Res 2002 30 17 3894 3900 10.1093/nar/gkf493 12202775 PMC137415

[B19] Capriotti E Fariselli P Rossi I Casadio R A three-state prediction of single point mutations on protein stability changes BMC Bioinformatics 2008 9 Suppl 2 S6 10.1186/1471-2105-9-S2-S6 18387208 PMC2323669

[B20] Krishnan VG Westhead DR A comparative study of machine-learning methods to predict the effects of single nucleotide polymorphisms on protein function Bioinformatics 2003 19 17 2199 2209 10.1093/bioinformatics/btg297 14630648

[B21] Wainreb G Ashkenazy H Bromberg Y Starovolsky-Shitrit A Haliloglu T Ruppin E Avraham KB Rost B Ben-Tal N MuD: an interactive web server for the prediction of non-neutral substitutions using protein structural data Nucleic Acids Res 2010 38 Suppl W523 528 10.1093/nar/gkq528 PMC2896130 20542913

[B22] Ye ZQ Zhao SQ Gao G Liu XQ Langlois RE Lu H Wei L Finding new structural and sequence attributes to predict possible disease association of single amino acid polymorphism (SAP) Bioinformatics 2007 23 12 1444 1450 10.1093/bioinformatics/btm119 17384424

[B23] Capriotti E Fariselli P Casadio R A neural-network-based method for predicting protein stability changes upon single point mutations Bioinformatics 2004 20 Suppl 1 I63 I68 10.1093/bioinformatics/bth928 15262782

[B24] Capriotti E Fariselli P Calabrese R Casadio R Predicting protein stability changes from sequences using support vector machines Bioinformatics 2005 21 Suppl 2 ii54 ii58 10.1093/bioinformatics/bti1109 16204125

[B25] Parthiban V Gromiha MM Schomburg D CUPSAT: prediction of protein stability upon point mutations Nucleic Acids Res 2006 34 Web Server issue W239 242 16845001 10.1093/nar/gkl190 PMC1538884

[B26] Zhou H Zhou Y Distance-scaled, finite ideal-gas reference state improves structure-derived potentials of mean force for structure selection and stability prediction Protein Sci 2002 11 11 2714 2726 12381853 10.1110/ps.0217002 PMC2373736

[B27] Bao L Zhou M Cui Y nsSNPAnalyzer: identifying disease-associated nonsynonymous single nucleotide polymorphisms Nucleic Acids Res 2005 33 Web Server issue W480 482 15980516 10.1093/nar/gki372 PMC1160133

[B28] Dobson RJ Munroe PB Caulfield MJ Saqi MA Predicting deleterious nsSNPs: an analysis of sequence and structural attributes BMC Bioinformatics 2006 7 217 10.1186/1471-2105-7-217 16630345 PMC1489951

[B29] Yue P Melamud E Moult J SNPs3D: candidate gene and SNP selection for association studies BMC Bioinformatics 2006 7 166 10.1186/1471-2105-7-166 16551372 PMC1435944

[B30] Ng PC Henikoff S SIFT: Predicting amino acid changes that affect protein function Nucleic Acids Res 2003 31 13 3812 3814 10.1093/nar/gkg509 12824425 PMC168916

[B31] Bao L Cui Y Prediction of the phenotypic effects of non-synonymous single nucleotide polymorphisms using structural and evolutionary information Bioinformatics 2005 21 10 2185 2190 10.1093/bioinformatics/bti365 15746281

[B32] Betz SF Disulfide bonds and the stability of globular proteins Protein Sci 1993 2 10 1551 1558 10.1002/pro.5560021002 8251931 PMC2142256

[B33] Waters ML Aromatic interactions in peptides: impact on structure and function Biopolymers 2004 76 5 435 445 10.1002/bip.20144 15478139

[B34] Care MA Needham CJ Bulpitt AJ Westhead DR Deleterious SNP prediction: be mindful of your training data! Bioinformatics 2007 23 6 664 672 10.1093/bioinformatics/btl649 17234639

[B35] Berman H Henrick K Nakamura H Markley JL The worldwide Protein Data Bank (wwPDB): ensuring a single, uniform archive of PDB data Nucleic Acids Res 2007 35 Database issue D301 303 17142228 10.1093/nar/gkl971 PMC1669775

[B36] Altschul SF Madden TL Schaffer AA Zhang J Zhang Z Miller W Lipman DJ Gapped BLAST and PSI-BLAST: a new generation of protein database search programs Nucleic Acids Res 1997 25 17 3389 3402 10.1093/nar/25.17.3389 9254694 PMC146917

[B37] Kabsch W Sander C Dictionary of protein secondary structure: pattern recognition of hydrogen-bonded and geometrical features Biopolymers 1983 22 12 2577 2637 10.1002/bip.360221211 6667333

[B38] Pei J Grishin NV AL2CO: calculation of positional conservation in a protein sequence alignment Bioinformatics 2001 17 8 700 712 10.1093/bioinformatics/17.8.700 11524371

[B39] Thomas PD Kejariwal A Coding single-nucleotide polymorphisms associated with complex vs. Mendelian disease: evolutionary evidence for differences in molecular effects Proc Natl Acad Sci U S A 2004 101 43 15398 15403 10.1073/pnas.0404380101 15492219 PMC523449

[B40] Kaminker JS Zhang Y Waugh A Haverty PM Peters B Sebisanovic D Stinson J Forrest WF Bazan JF Seshagiri S Distinguishing cancer-associated missense mutations from common polymorphisms Cancer Res 2007 67 2 465 473 10.1158/0008-5472.CAN-06-1736 17234753

[B41] Boyle EI Weng S Gollub J Jin H Botstein D Cherry JM Sherlock G GO::TermFinder--open source software for accessing Gene Ontology information and finding significantly enriched Gene Ontology terms associated with a list of genes Bioinformatics 2004 20 18 3710 3715 10.1093/bioinformatics/bth456 15297299 PMC3037731

[B42] Chang CC Lin CJ Training nu-support vector classifiers: theory and algorithms Neural Comput 2001 13 9 2119 2147 10.1162/089976601750399335 11516360

[B43] Baldi P Brunak S Chauvin Y Andersen CA Nielsen H Assessing the accuracy of prediction algorithms for classification: an overview Bioinformatics 2000 16 5 412 424 10.1093/bioinformatics/16.5.412 10871264

